# Factors to Consider in the Use of Vital Signs Wearables to Minimize Contact With Stable COVID-19 Patients: Experience of Its Implementation During the Pandemic

**DOI:** 10.3389/fdgth.2021.639827

**Published:** 2021-09-20

**Authors:** Esther Monica Pei Jin Fan, Shin Yuh Ang, Ghee Chee Phua, Lee Chen Ee, Kok Cheong Wong, Franklin Chee Ping Tan, Lydia Wan Har Tan, Tracy Carol Ayre, Chee Yong Chua, Benedict Wee Bor Tan, Khung Keong Yeo

**Affiliations:** ^1^Nursing Division, Singapore General Hospital, Singapore, Singapore; ^2^Department of Respiratory and Critical Care Medicine, Singapore General Hospital, Singapore, Singapore; ^3^Organisational Transformation, SingHealth, Singapore, Singapore; ^4^Nursing Division, Changi General Hospital, Singapore, Singapore; ^5^Office for Service Transformation, SingHealth, Singapore, Singapore; ^6^Office of Innovation, Changi General Hospital, Singapore, Singapore; ^7^Emerging Services and Capabilities Group, Integrated Health Information Systems, Singapore, Singapore; ^8^Division of Digital Strategy, SingHealth, Singapore, Singapore; ^9^Department of Cardiology, National Heart Centre Singapore, Singapore, Singapore; ^10^Duke-National University of Singapore Medical School, Singapore, Singapore

**Keywords:** COVID-19, vital signs wearables, vital signs monitoring, digital health, digital solution

## Abstract

The COVID-19 pandemic has created a huge burden on the healthcare industry worldwide. Pressures to increase the isolation healthcare facility to cope with the growing number of patients led to an exploration of the use of wearables for vital signs monitoring among stable COVID-19 patients. Vital signs wearables were chosen for use in our facility with the purpose of reducing patient contact and preserving personal protective equipment. The process of deciding on the wearable solution as well as the implementation of the solution brought much insight to the team. This paper presents an overview of factors to consider in implementing a vital signs wearable solution. This includes considerations before deciding on whether or not to use a wearable device, followed by key criteria of the solution to assess. With the use of wearables rising in popularity, this serves as a guide for others who may want to implement it in their institutions.

## Introduction

A pneumonia of unknown cause was detected in Wuhan, and was first reported to World Health Organization (WHO) on 31st December (1). The disease spread quickly and was soon characterized by WHO as a pandemic on 11th March 2020 (2). The first case of COVID-19 infection in Singapore was detected on 23 January 2020. By 18 November, the number of cases has risen to 58,135, with 28 fatalities (3). Consequently, the healthcare industry met with various challenges. The need for healthcare facilities and healthcare workers (HCWs) rose rapidly (4). Demand for equipment, personal protective equipment (PPE), medications, and consumables rose so quickly that supply chains struggled to meet them.

With rising cases of COVID-19, facilities were converted/created to care for them (4). There was a need to monitor more patients with less HCWs while preserving PPE. It was also necessary to ensure that healthcare remains cost effective. Additionally, easing the strain on HCWs to avoid burnout was a major consideration given the extended duration of this pandemic. Exploration of the use of vital signs wearables, which begun a few years ago, was accelerated during this period in attempt to meet these needs.

This paper presents an overview of factors to consider in implementing a vital signs wearable solution during an infectious disease outbreak. In the age where the use of wearables is expected to rise, these learnings may prove useful for those implementing them in the future.

## Purpose and Suitability of Wearables

Vital signs wearables are devices worn for continuous and non-invasive monitoring of vital signs (5). Once attached to the patient, remote and tetherless monitoring occurs (5), reducing the contact of nurses with infectious cases and reducing the workload of performing vital signs measurements manually.

**Figure 1:**
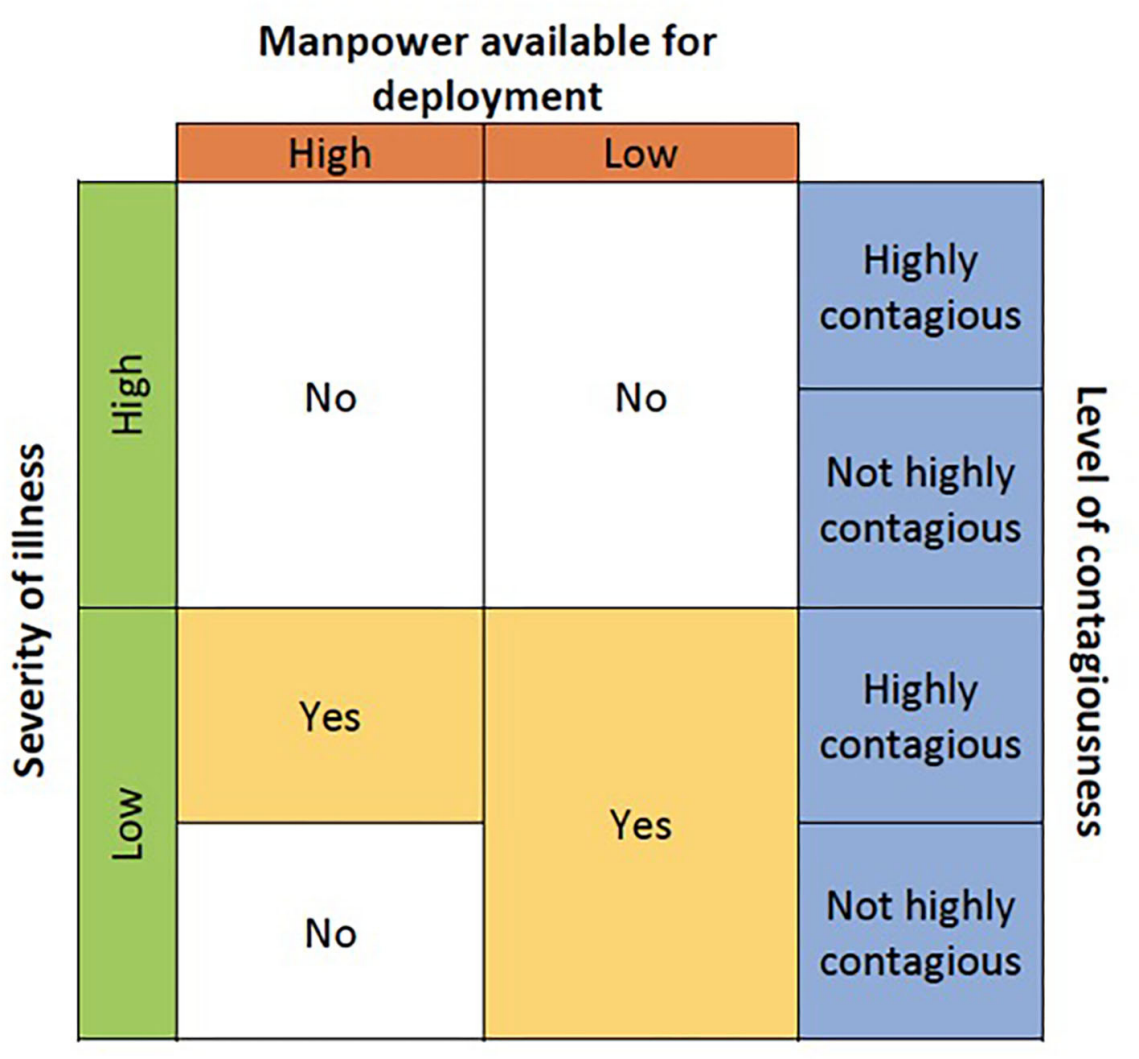
Decision on whether a vital signs wearable is suitable.

Before deciding which wearable device to use, the purpose and suitability of wearables in the specific clinical environment should be considered. This depends on the severity and contagiousness of disease, as well as availability of manpower. Contagiousness of disease refers to how easily it spreads. It is influenced by multiple factors including but not limited to the infectious period, mode of transmission, and ability of the pathogen to survive outside of a host. Refer to Figure 1 for a decision guide on the suitability of wearables.

Patients with higher severity of illness are unlikely to be highly mobile. Hence traditional bedside monitors rather than wearables may be more suited. Traditional bedside monitors are not affected by poor WIFI/Bluetooth signal strength [a common limitation for wearables (6)], this is a more reliable form of monitoring for patients requiring close monitoring.

For patients with mild illness, wearables may be considered as they are mobile (6). In a situation with high manpower and no contagious disease, spot monitors may suffice. If manpower is low with no contagious disease, wearables could be used for mass and remote monitoring, relieving nurses of the task of manually taking parameters.

If the disease is highly contagious, wearables could be deployed regardless of manpower availability to minimize patient-nurse contact, reducing the exposure of the nurse to the contagion while preserving PPE.

In our case, the use of wearables was in an isolation setting with low severity of illness. Patients were confirmed or suspected COVID-19 cases with low risk for complications. They presented with mild symptoms, had no other medical conditions and could independently perform activities of daily living (ADLs). Due to the increased need for nurses as well as the expected mild illness, the nurse: patient ratio in our setting was lower than that of a general ward. Additionally, COVID-19 is highly contagious. Hence, the decision was made to use wearables with the purpose of patient monitoring while minimizing nurse-patient contact and to preserve PPE, not for early detection of deterioration.

## Criteria to Determine Suitability of the Wearable Vital Signs Monitoring Solution

**Figure 2:**
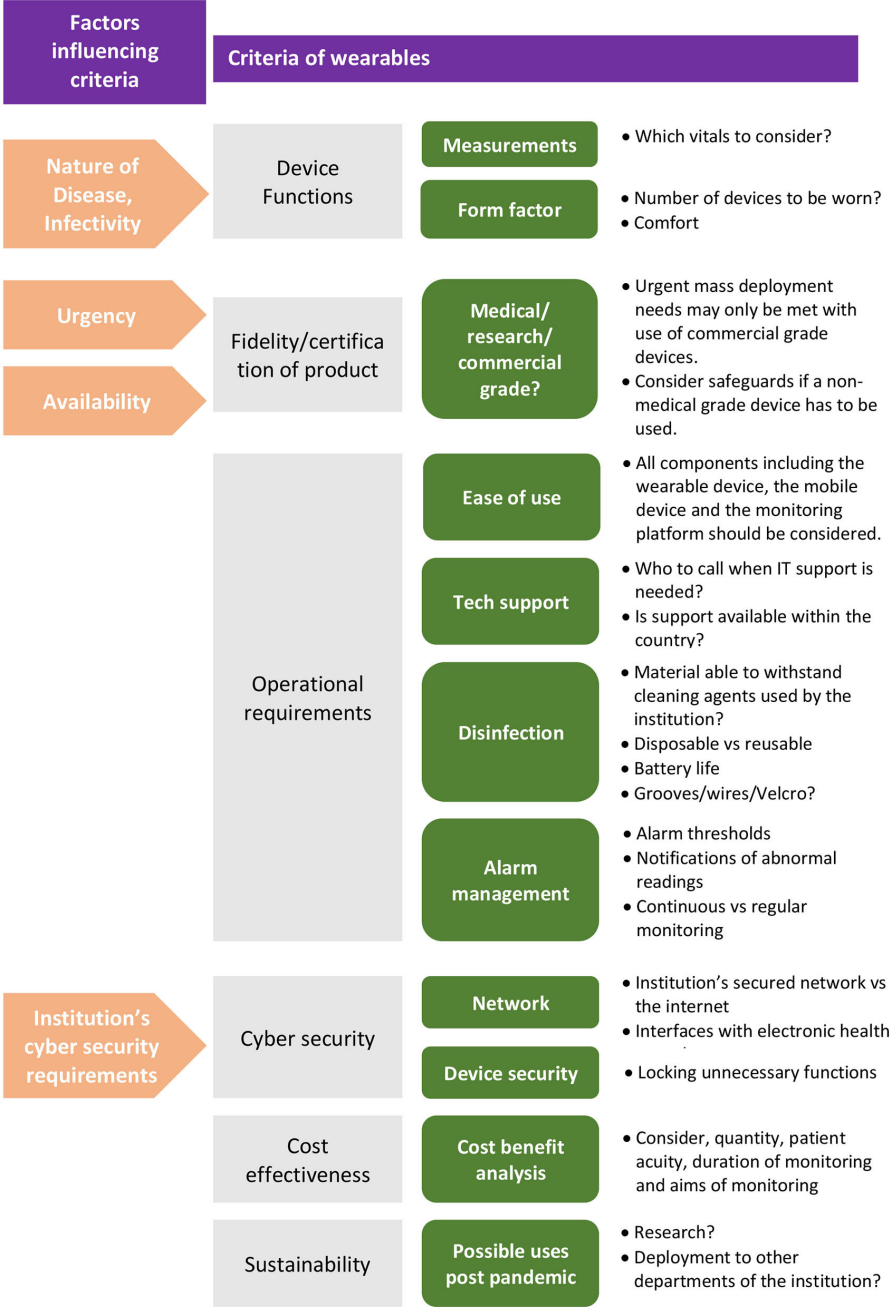
Overview of criteria to determine suitability of a wearable vital signs monitoring solution.

Once decided that a wearable solution is suitable, a myriad of factors influence the selection of the specific solution. Key criteria to consider are: device functions, fidelity of the product, operational requirements, cyber security, cost effectiveness and sustainability. Refer to Figure 2 for an overview of the criteria involved.

### Device Functions

Device functions refers to the specifications of the device in terms of its physiological measures [e.g., heart rate (HR)], as well as its form factor. Required functions largely depend on the nature of disease.

#### Measurements

There are solutions for capturing full sets of vital signs: HR, respiration rate (RR), oxygen saturation (SpO_2_), temperature, and blood pressure (BP). An example would be the ViSi mobile by Sotera. However, it requires the patient to be strapped onto multiple devices which is not ideal (discussed further in the next section). Therefore, prioritization of vital signs is paramount.

In our case, as COVID-19 is a respiratory disease, monitoring of RR (7), and SpO_2_ is important for quick recognition of deterioration (8). HR is also essential as changes in HR occurs as a compensatory mechanism in the early stage of clinical deterioration (9). Therefore, HR, RR, and SpO_2_ were prioritized to require close monitoring and the solution we selected measured those parameters.

BP and temperature were measured for our patients at regular intervals (e.g., 4 hourly/6 hourly), rather than continuously. This is because BP is usually not the first vital sign to respond during a deterioration (9), and frequent temperature monitoring for adults with normal thermoregulation is usually not mandated (10). These measurements were timed to be performed when the nurse entered the room for other purposes. This could be done without compromising on patient safety as our patient population was at low risk for complications.

#### Form Factor

Wearable devices come in many forms including smart watches, chest patches, and pulse oximeters (11). An ideal wearable should have maximum functionality with minimum burden (12). In our population where patients were ADL independent, devices that do not restrict movement were preferred. Comfort of the device ensures compliance on the patients' part. An uncomfortable device may lead to frequent removal, adding burden on nurses to repeatedly troubleshoot the lack of vital signs readings.

**Figure 3:**
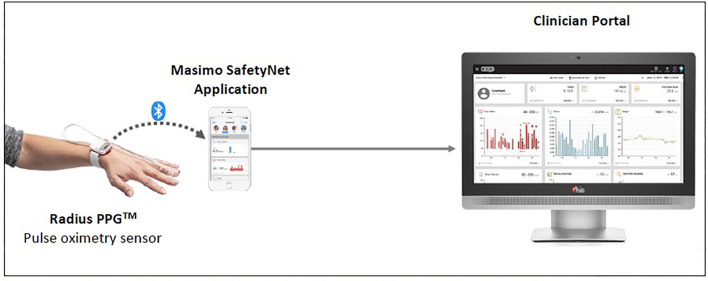
Masimo SafetyNetTM (1) (*Reproduced with permission from Masimo*).

Considering the prioritized measurements and form factor, the Masimo SafetyNet^TM^ solution was used in our setting (refer to Figure 3). The Radius PPG^TM^ senses the patient's vital signs. The readings are then reflected on the Masimo SafetyNet^TM^ application as well as on a clinician portal at the nurses' counter.

The Radius PPG^TM^ is designed to provide accurate pulse oximetry in the presence of motion and low perfusion (13). It also provides a RR derived from phlethysmography (13). An automated measurement of RR is beneficial as RR changes are seen early in deterioration, yet it is often deemed least important by nurses and it is tedious to manually count it (9). The Radius PPG^TM^ is light weight. In a survey of 37 patients, 83.8% agreed or strongly agreed that it was comfortable to wear, and 89.2% agreed or strongly agreed that it did not restrict their movement.

One limitation of the Radius PPG^TM^ was that it had to be removed before a shower. However, it was easy for patients to replace it afterwards following the instructions on a poster provided.

### Fidelity of the Product

Wearables available in the market range from commercial grade to medical grade to research grade (11). A device is considered medical grade if it fulfills the regulatory requirements of the region where it is used. For instance, Food and Drug Administration (FDA) in the United States which evaluates effectiveness of the device and its risk for harm (14), European CE mark that affirms the device meets high safety, health and environmental protection requirements (14) and Health Sciences Authority in Singapore.

#### Duration to Implementation

For use in a healthcare setting, a medical grade device is required. As time is required for validation of new devices as well as for obtaining regulatory requirements, quick deployment during a pandemic demands for wearables that are medical grade. As a note of caution, devices marketed to be medical grade may only have some (not all) of their parameters clinically validated. For instance, Everion by Biofourmis is marketed to be medical grade (15). However, only HR and SpO_2_ are clinically validated vital signs while heart rate variability and RR are not (15). Care should be taken to ensure all vital signs prioritized by the medical team have been clinically validated.

#### Availability of Supply

Surges in demand for medical devices coupled with supply chain disruptions caused some medical grade devices to be unavailable. For example, Canada faced a supply mismatch in pulse oximeters during this pandemic (16). In some cases, a commercial grade device may be deployed due to the lack of a better option. In such cases, a safety net should be in place. The institution should make available some medical grade devices (not necessarily wearables) for rechecking purposes if the patient's vital signs were recorded to be out of range on the commercial grade device, or if the patient reports to be unwell.

### Operational Requirements

#### Ease of Use

To facilitate training and prevent errors, the solution should be simple. It should enhance the workflow instead of creating an additional burden.

Some practical questions for considerations are as follows:

- Is the wearable device easy to apply?
- Can the patient easily reapply it if it has to be removed for a shower?
- Is the monitoring dashboard clear? Can it be customized?
- How are notifications of abnormal vital signs displayed?
- Is there a sound to alert nurses of an abnormal vital sign?
- Is the monitoring dashboard viewed from an existing intranet environment or will it require separate devices connected to the internet for viewing? (Each of these decisions will require its own cybersecurity assessment).

#### IT Support

A support structure should be emplaced. Nurses should have ready access to help when technical difficulties are faced. The IT support should consist of staff within the hospital (who can respond quickly), as well as personnel from the company (who will be able to troubleshoot more technical issues). Use of products with a local support office is preferred.

It is also advisable to involve the IT and informatics team from the start of the project.

In our implementation, a common reason for troubleshooting was that the vital signs were not reflected on the clinician portal. Initially, the most common reason was that the battery of the Radius PPG^TM^ ran out. Subsequently, we learnt that if the mobile device was not in use for a prolonged duration, the Bluetooth of the mobile device goes to sleep cutting off the connection between the sensor and the mobile device. This was the main limitation experienced during this implementation as the nurse would need to enter the room to turn on the application in order to continue monitoring the patient. Our team was informed that all current mobile devices turns off Bluetooth after prolonged inactivity. Hence, this is a limiting factor to consider for the use of any wearables relying on Bluetooth connection to a mobile device till future developments resolves this.

#### Disinfection

The device has to withstand disinfection procedures as per institution's guidelines. In our institution, disinfection with Ultraviolet (UV) treatment or Hydrogen Peroxide Vaporization (HPV) is required for areas or items used by patients who are COVID-19 positive to prevent cross contamination.

Wearables may be disposable or reusable (with rechargeable batteries/disposable batteries). Reusable wearables need to be removed from the room for charging at regular intervals. However, if the patient is not discharged by then, the device which have not undergone UV or HPV treatment cannot be removed from the room for charging. Hence, disposable wearables are preferred. Disposable wearables vary in their battery life. A longer battery life reduces frequency at which they need to be replaced. However, the battery life should not be much longer than the expected length of stay to minimize waste.

If reusable devices are used, disposable batteries would be preferred over rechargeable batteries for the same reason mentioned above. Ease of cleaning should also be considered. Wireless devices without grooves and without materials difficult to disinfect (e.g., Velcro) would be preferred.

For the Masimo SafetyNet^TM^ solution, the Radius PPG^TM^ is disposable with a reusable chip. In our setting, the reusable chip was wiped with 70% isopropyl alcohol (as per manufacturer's instructions) and undergone UV treatment as per our institution's requirements. It was a small chip without many grooves and it was easy to clean.

#### Alerts and Alarms

A platform displaying each patient's vital signs at a remote location (e.g., the nurses' station) will be beneficial. The platform should alert nurses to any abnormalities.

Safeguards must be in place to ensure that no deteriorating patient is undetected. Customisable alarm thresholds are necessary to prevent unacceptably high number of alarms (17), preventing alarm fatigue. Customisable dashboards to support operational processes will also be beneficial.

Alarm management is challenging when continuously monitoring patients who are ADLs independent. Traditional vital signs thresholds were set for vital signs taken at rest. However, patients who are ADLs independent may be moving or talking causing artifacts which are one of the biggest problems in data evaluation (5). Although some studies suggest that continuous monitoring with automated alerts improves patient outcomes (6), alarm fatigue could be counter-productive. To prevent alarm fatigue, patients who are relatively well with low risk for complications should have regular rather than continuous monitoring.

Even though most wearable solutions offer continuous monitoring, the purpose for wearables in our situation was not meant for that purpose. As mentioned, our aim was to minimize contact between nurses and patients. Therefore, staff should not be additionally burdened to continuously monitor the patients just because the wearables are able to do so. Rather, adjustments to work processes should be made to maximize the benefits of technology without increasing the burden on staff. For instance, protocol may require nurses to check the wearables recordings at fixed intervals rather than continuously.

### Cyber Security

Cyber security is the practice of defending computers, servers, mobile devices, electronic systems, networks, and data from malicious attacks. As healthcare information are highly sensitive, confidentiality is paramount. All patient identifiers and health information should be protected (18). Therefore, the implementation of the wearables necessitates thefollowing:

- Device security of any mobile devices that are used to collect, store, or transmit information;
- Secure data transmission and storage- Data transmission and data at rest have to follow relevant security guidelines (e.g., Health Security Instruction Manual). Data stored in the cloud has to be anonymised to reduce exposure risks of 3rd party product (18).
- Proper account provisioning and management; patient re-identification governance process; data backup and device fidelity are also important hygiene considerations.

Other important risks include malicious hacking to corrupt or alter data collected, introduction of malware that impairs the performance of the device, or the devices being used as portals or mediums for cyber criminals to gain access to enterprise digital assets such as the Electronic Medical Records (EMR) system.

In our institution, EMR and other enterprise IT systems are connected to a private, secured network, not the Internet, as governed by the public healthcare IT policies. Ideally, the wearables solution should sit within this secured network for enhanced cybersecurity and work processes. If the solution was within the secured network and integrated with the EMR, readings from the wearables would be directly charted into the EMR without transcription errors or additional effort from nurses. In addition, full patient identification (e.g., name and registration number) may be viewed for easy patient identification.

However, most wearable solutions are designed to store data in a public cloud. Hence, they require internet access. Furthermore, time is required to architect a secured solution to interface data from the wearables solution to the EMR system. These reasons ruled out our preference of sitting the wearable system in the secured network.

Working with our Chief Information Security Officer (CISO) and IT teams, we arrived at a quick implementation of an internet enabled solution. To maintain cyber security, the wearables solution was a stand-alone system with no patient identifiers within it. Pseudo IDs were used to mitigate risks associated with cybersecurity. All functions in the mobile device except those required for the solution to work were locked down to prevent usage habits from sabotaging security of the device or software system.

Another possible scenario without syncing the wearables solution with the EMR systems would be for vital signs to be measured and self-charted by patients onto a platform that can be accessed by the nurses. This is not recommended as there are some major limitations. A similar approach was carried out in some community isolation facilities (CIFs) in Singapore. CIFs isolated patients with very mild symptoms not requiring hospital stay. These patients were provided with vital signs monitoring devices (not wearables) and were required to self-chart their vital signs. Challenges faced were that some patients confused the PR with SpO_2_ and entered “PR = 99 bpm, SpO_2_ = 60%,” instead of the other way round. Patients may also measure their vital signs after physical activity, leading to a high number of false alerts being sent to clinicians.

### Cost Effectiveness

It is unclear when the pandemic will end. Hence, the solution needs to be cost effective. Severity of illness, quantity required, aims of monitoring using the wearables should be taken into consideration in determining its cost effectiveness.

### Sustainability

To prevent wastage, potential uses of the wearables after the pandemic should be contemplated during the selection of the solution. Suggestions for future use of wearables would be for research purposes or for feasibility trials in the management of other groups of patients (such as outpatients or patients enrolled in a hospital at home program). If the use of that wearable device proves successful, plans could be made to integrate the wearables system with the EMR within the secured network, and to implement its use across the institution.

## Conclusion

The use of vital signs wearables can be expected to rise with the ongoing advancement in technology. Although this list of considerations is not exhaustive, this may be a starting point for those looking to implement a wearables solution in their area.

## Data Availability Statement

The original contributions presented in the study are included in the article/supplementary material, further inquiries can be directed to the corresponding author/s.

## Author Contributions

EF, GP, BT, and KY were involved in the implementation of the solution with support from all other authors. EF, LT, LC, CC, and BT were involved in the drafting of the manuscript. SA and KY provided mentorship in the drafting of the final manuscript and supervised the project. All authors were involved in the conception of the original idea.

## Conflict of Interest

KY declares the following conflicts of interest: 1. Research funding, unrelated to this project: Biofourmis, Holmusk, Bayer, Medtronic, Astra Zeneca, and Shockwave Medical. 2. Consultancy: Abbott Vascular, Boston Scientific, Medtronic, Amgen, Bayer, Novartis, and Medopad. 3. Speaker or Honararia: Shockwave Medical, Abbott Vascular, Boston Scientific, Medtronic, Philips, Alvimedica, Biotronik, Amgen, Astra Zeneca, Orbus Neich, and Bayer. The remaining authors declare that the research was conducted in the absence of any commercial or financial relationships that could be construed as a potential conflict of interest.

## Publisher's Note

All claims expressed in this article are solely those of the authors and do not necessarily represent those of their affiliated organizations, or those of the publisher, the editors and the reviewers. Any product that may be evaluated in this article, or claim that may be made by its manufacturer, is not guaranteed or endorsed by the publisher.

## References

[1] World Health Organization. Rolling Updates on Coronavirus Disease (COVID-19). (2020). Available online at: https://www.who.int/emergencies/diseases/novel-coronavirus-2019/events-as-they-happen

[2] World Health Organization. WHO Timeline- Covid-19. (2020). Available online at: https://www.who.int/news-room/detail/27-04-2020-who-timeline---covid-19

[3] Ministry of Health Singapore. Updates on COVID-19 (Coronavirus Disease 2019) Local Situation. (2020). Available online at: https://www.moh.gov.sg/covid-19

[4] Fan PEM Fazila A Ang SY Elena BMA Norhayati BA Chiang JL. Preparation and response to COVID-19 outbreak in Singapore: a case report. Infection Dis Health. (2020) 25:216–8. 10.1016/j.idh.2020.04.002 32402779 PMC7203022

[5] Jacobsen M Dembek TA Kobbe G Gaidzik PW Heinemann L. Noninvasive continuous monitoring of vital signs with wearables: fit for medical use? J Diabet Sci Technol. (2021) 15:34–43. 10.1177/1932296820904947 32063034 PMC7783016

[6] Leenen JP Leerentveld C van Dijk JD van Westreenen HL Schoonhoven L Patijn GA. Current evidence for continuous vital signs monitoring by wearable wireless devices in hospitalized adults: systematic review. J Med Internet Res. (2020) 22:e18636. 10.2196/18636 32469323 PMC7351263

[7] Quaresima V Ferrari M. More on pulse oximetry for monitoring patients with COVID-19 at home. Ann Am Thoracic Soc. (2020) 17:1496. 10.1513/AnnalsATS.202006-701LE 32866031 PMC7640719

[8] Luks AM Swenson ER. Pulse oximetry for monitoring patients with COVID-19 at home. potential pitfalls and practical guidance. Ann Am Thoracic Soc. (2020) 17:1040. 10.1513/AnnalsATS.202005-418FR 32521167 PMC7462317

[9] Mok W Wang W Cooper S Ang ENK Liaw SY. Attitudes towards vital signs monitoring in the detection of clinical deterioration: scale development and survey of ward nurses. Int J Qual Health Care. (2015) 27:207–13. 10.1093/intqhc/mzv019 25888564

[10] Cahill K. Royal Prince Alfred Hospital Patient Observation (Vital Signs) Policy-Adult. Sydney South West Area Health Service (2014). Available online at: https://support.biofourmis.com/hc/en-us/articles/212369329-Everion-data-quality-

[11] Dunn J Runge R Snyder M. Wearables and the medical revolution. Personalized Med. (2018) 15:429–48. 10.2217/pme-2018-0044 30259801 PMC12294383

[12] Bodine K Gemperle F. Effects of functionality on perceived comfort of wearables. In: Seventh IEEE International Symposium on Wearable Computers, 2003. Proceedings. Toronto, ON: Citeseer (2003). p. 57.

[13] Masimo. Masimo SafetyNet. Alexandria, VA (2021).

[14] Conley D. Two Paths for Medical Device Approval: FDA vs. CE. Health Management. New York, NY: AnnalsATS (2015).

[15] Biofourmis. Everion Data Quality. New York, NY (2021).

[16] Government of Canada. Medical Device Shortage: Saturometer (Pulse Oximeter). England (2020).

[17] Downey C Randell R Brown J Jayne DG. Continuous versus intermittent vital signs monitoring using a wearable, wireless patch in patients admitted to surgical wards: pilot cluster randomized controlled trial. J Med Internet Res. (2018) 20:e10802. 10.2196/10802 30538086 PMC6305881

[18] Izmailova ES Wagner JA Perakslis ED. Wearable devices in clinical trials: hype and hypothesis. Clin Pharmacol Therapeut. (2018) 104:42–52. 10.1002/cpt.966 29205294 PMC6032822

